# Supplementary material for: Integrated and efficient diffusion-relaxometry using ZEBRA
Source: Sci Rep. 2018 Oct 11;8:15138. doi: 10.1038/s41598-018-33463-2 (PMC6181938; doi:10.1038/s41598-018-33463-2)
Supplement: Supplementary file 1 — Supplementary Figures S1-S5 [file 41598_2018_33463_MOESM1_ESM.pdf]

Supporting Material for: Integrated and efficient diffusion-relaxometry using ZEBRA

Jana Hutter, Paddy J Slator, Daan Christiaens, Rui Pedro AG Teixeira, Thomas Roberts, Laurence Jackson, Anthony N Price, Shaihan Malik, Joseph V Hajnal

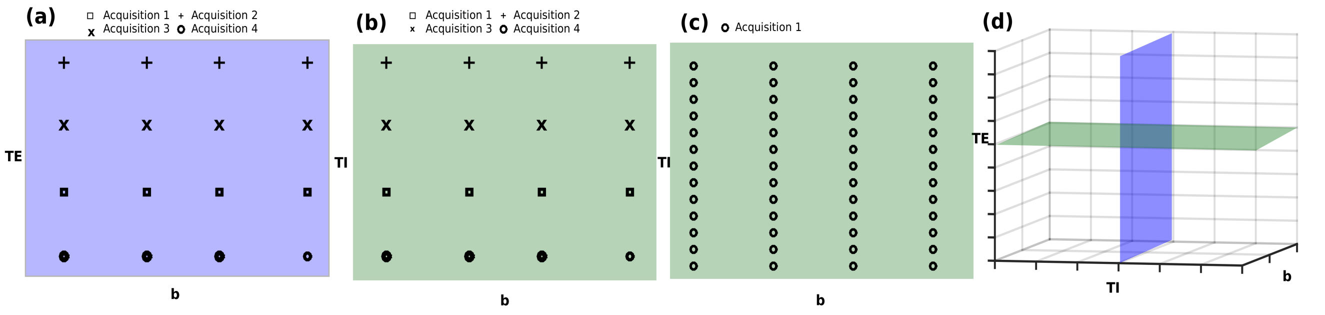

Supporting Figure S1: Sampling schemes of the multidimensional acquisition parameter space. (a) Scheme of conventional T2-diffusion acquisitions such as e.g. Veraart et al., sampling the TE-b domain with individual diffusion acquisitions for each considered echo time. (b) Scheme of conventional T1-diffusion acquisitions with separate diffusion acquisitions per TI such as e.g. DeSantis at al. (c) Scheme as employed by T1-diffusion studies using slice-shuffling approaches to sample the TI-diffusion domain in one scan. (d) Depiction of the two considered combinations T1-diffusion and T2-diffusion as planes within the larger three-dimensional TI-TE-b space.

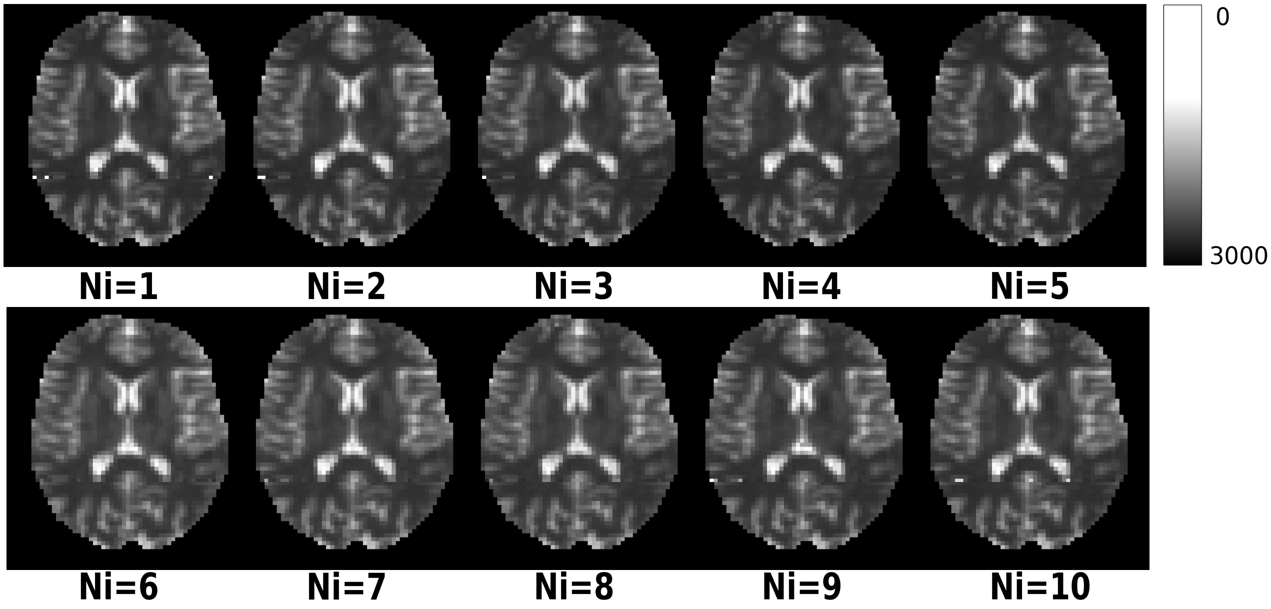

Supporting Figure S2: Results from the adult experiment sampled with 27 TIs and sub-sampled at $N_i = 1, .., 10$ are illustrated. A mid-brain slice of the obtained T1 maps is depicted for all interleave factors - resulting in 27 (interleave 1) to 3 (interleave 10) TI points - is shown.

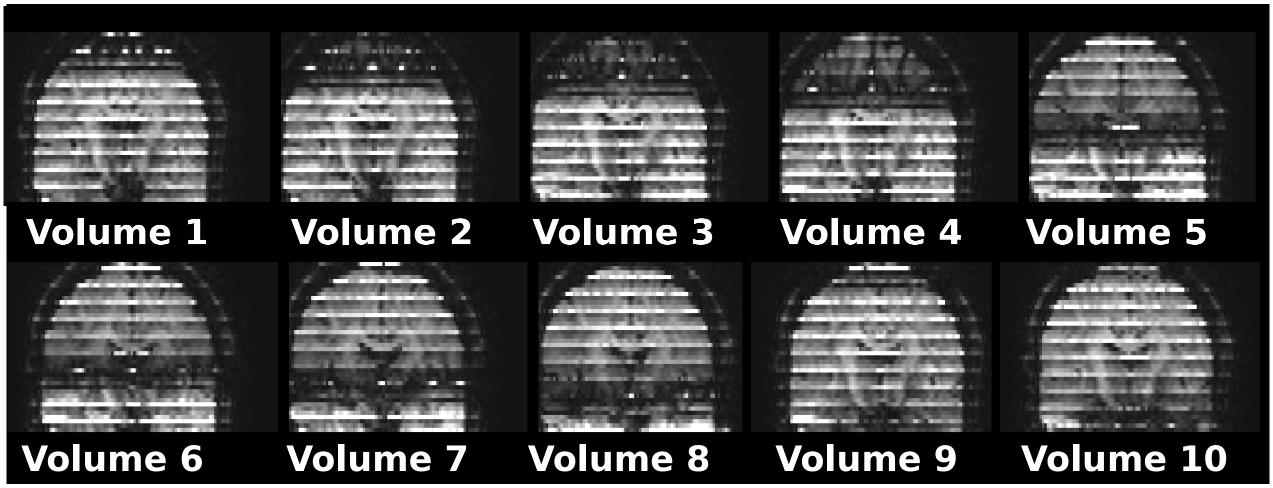

Supporting Figure S3: The volumes from one superblock with N_s/N_i = 40/4=10 are illustrated in coronal orientation before sorting to volumes, showing the varying TI and diffusion encodings per slice.

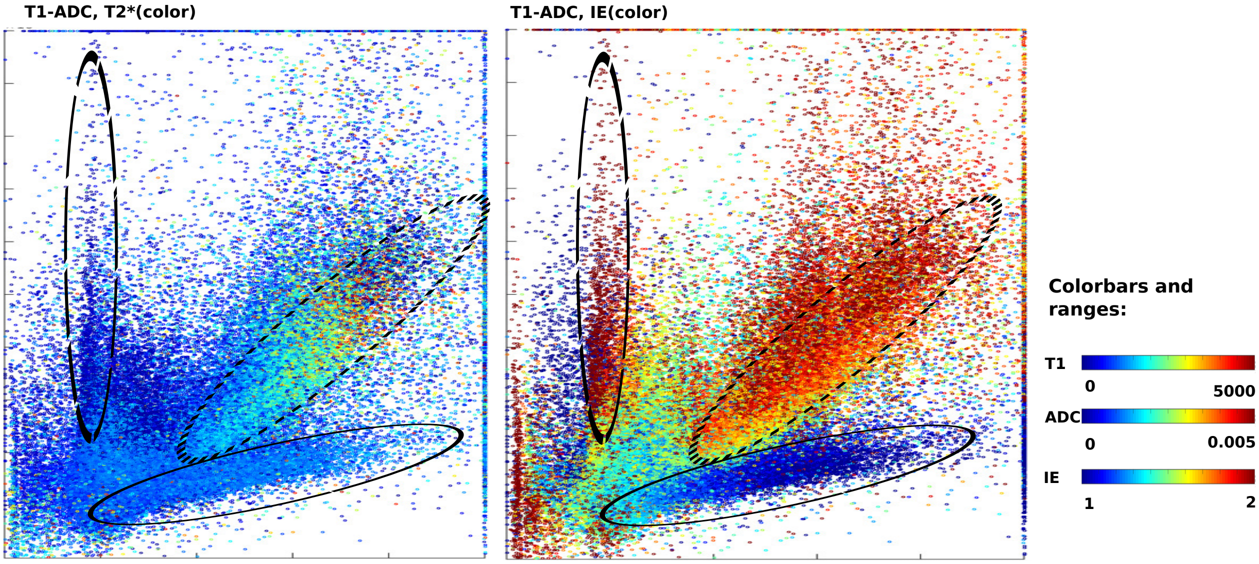

Supporting Figure S4: Scatter plot from the parameter maps obtained from all voxels within the brain mask, displayed in T1 (x-axis) vs. ADC (y-axis) space. The color in the left subfigure is defined by T2*, in the right subfigure by IE. The three added circles draw the attention to three regions with different correlation of the described parameters: region 1 (marked by continuous circle), region 2 (marked by dashed circle) and region 3 (marked by dotted circle).

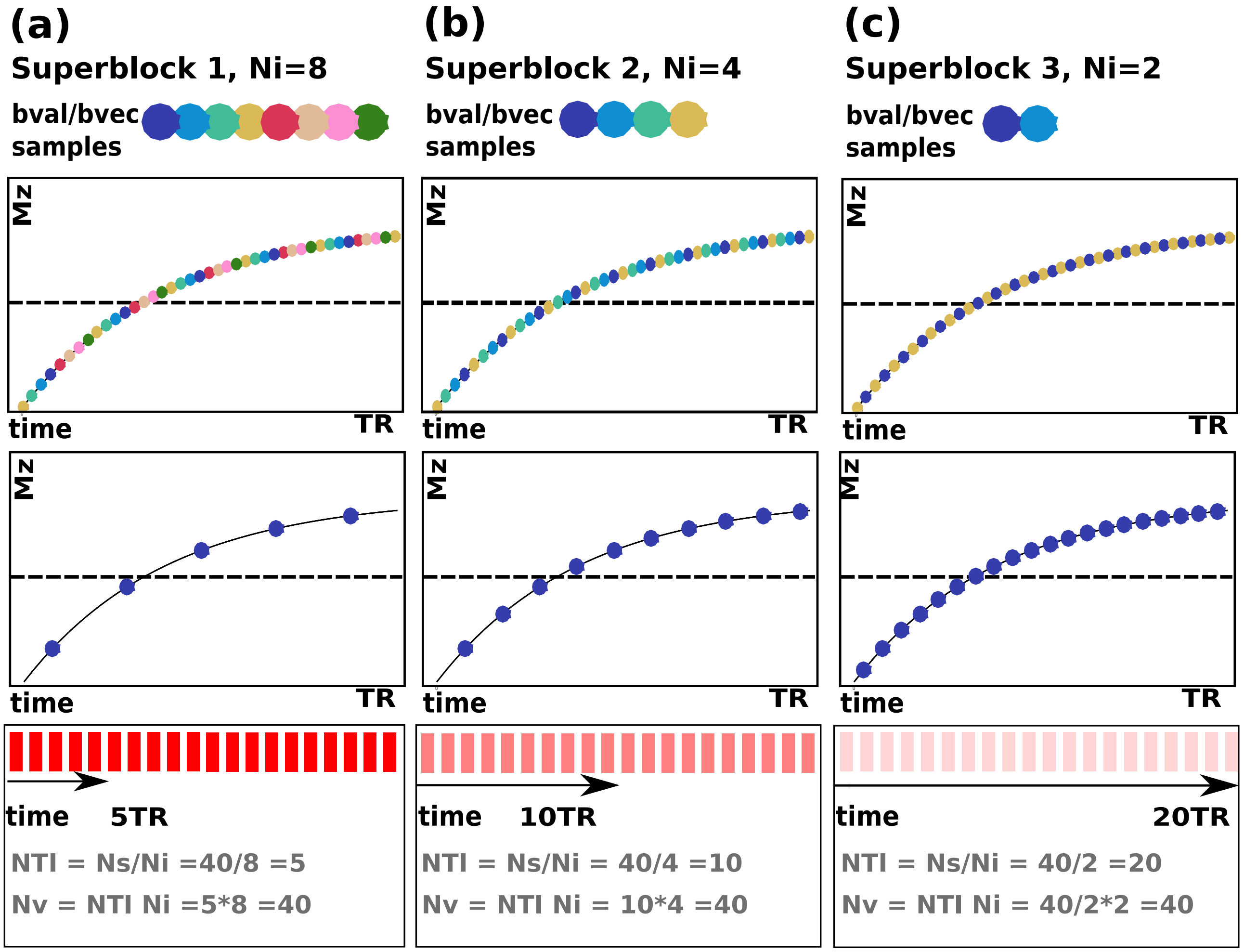

Supporting Figure S5: The sequence flexibly accommodates superblocks with various interleaving factors N_i. For 3 different settings of N_i, (a) 8, (b) 4 and (c) 2, the sampling is illustrated schematically in the top row, the sampling density for individual b-values is illustrated in the middle row and the required volumes are depicted in the lower row.
